# Description of a new species of *Alburnus* Rafinesque, 1820 (Actinopterygii, Cyprinidae, Leuciscinae) from the Kolpa River in the Sava River system (upper Danube drainage), with remarks on the geographical distribution of shemayas in the Danube

**DOI:** 10.3897/zookeys.688.11261

**Published:** 2017-08-09

**Authors:** Nina G. Bogutskaya, Primož Zupančič, Dušan Jelić, Oleg A. Diripasko, Alexander M. Naseka

**Affiliations:** 1 Naturhistorisches Museum Wien, Burgring 7, Vienna 1010, Austria; 2 Dinaric Research Institute, Dolsko, 14, 1262 Slovenia; 3 Croatian Institute for Biodiversity, Lipovac I, no. 7, HR-10000 Zagreb, Croatia; 4 Institute of Fisheries and Marine Ecology (IFME), Berdyansk, Ukraine; 5 Department of Ichthyology and Hydrobiology, Faculty for Biology and Soil, Saint Petersburg State University, 7/9 Universitetskaya nab., Saint Petersburg, 199034 Russia

**Keywords:** Freshwater and anadromous shemayas, taxonomy, morphology, *Alburnus sarmaticus*, *Alburnus mento*, Black Sea-Sea of Azov basin

## Abstract

*Alburnus sava*, new species, is described from the Kolpa River. The Kolpa is a tributary of the Sava, a major tributary of the Danube River, in the Black Sea basin. *Alburnus sava* is distinguished from its congeners in the Danube drainage, *A. mento* and *A. sarmaticus*, by having 23−27, usually 24−26, gill rakers; the ventral keel usually completely covered by scales (scaleless part maximum 15% of the keel length); 15−16, mode 15, branched pectoral-fin rays; the length of the gill raker at the junction of the arch limbs 65−70% of the length of the opposite outer gill filament; and a relatively long lower jaw (37−40% HL or 112−130% interorbital width). *Alburnus sava* is a large-sized potamadromous shemaya known to occur in the entire Sava drainage. The taxonomic status of *A. mento* and *A. sarmaticus* is confirmed. *Alburnus danubicus* is discussed and as there are no new arguments, it is kept as a valid species. New details on the distribution of shemayas in the Danube drainage are presented.

## Introduction

Shemaya is a Russian vernacular name based on the Persian name *shah-mahi*, King’s fish, used for *A. chalcoides* Gueldenstaedt, 1772 in the Caspian Sea basin. It is also commonly applied to a number of nominal species and subspecies in the Caspian and the Black seas since Borodin (1904) and Berg (1905, 1916). Shemayas were later assigned to a distinct genus *Chalcalburnus* Berg, 1932 and discriminated from *Alburnus* by a usually scaled (almost completely or completely covered by scales) ventral keel between the pelvic fins and the anus (vs. usually scaleless in *Alburnus*) and an increased number of lateral-line scales, usually over 55 (vs. usually less then 55 in *Alburnus*, with the exception of *A. filippii* Kessler, 1877 with up to 63) (Berg 1932, 1933). Recent morphological studies (Freyhof and Kottelat 2007a,b, Özuluğ and Freyhof 2007) supported the idea that shemayas form a distinct morphological group of species though included in the genus *Alburnus* Rafinesque, 1820. Not all shemaya species have been studied using molecular markers but preliminary data support the conclusion that they form a monophyletic group (Perea et al. 2010, Geiger et al. 2014).

Earlier authors (e.g., Kessler 1877, Berg 1916, 1923, 1932, 1933, 1949, Abdurakhmanov 1962, Tscherbukha 1965, Elanidze 1983, Movchan and Smirnov 1983) emphasized an extremely high similarity of shemayas in the few characters used in their discrimination − usually only the number of branched anal-fin rays and gill rakers, and some body measurements. For example, a reliable range of the branched anal-fin rays based on large samples examined of the Caspian shemaya is 12−17 (=12½−17½ according to the method of counting by Kottelat and Freyhof (2007) with means 14.6 (Berg 1932) and 14.7 (Abdurakhmanov 1962). The Black Sea forms are characterised by higher numbers of rays, averaging 15.6 in the Don drainage (Berg 1932), 15.9 in the South Bug (Tscherbukha 1965), 15.2 in the Berda River, Sea of Azov basin (Loshakov 1973) with a range of 14−17 (=14½−17½) (i.e. within the range in the Caspian shemaya). However, there are also local forms in the Black Sea basin with a lower number of branched anal-fin rays. For example, the Colchis (or Georgian) shemaya from Western Transcaucasia has a range of branched anal-fin rays of 12−16 (=12½−16½), usually 13−15, and a mode of 14 (Berg 1949, Elanidze 1983). However, later studies (Freyhof and Kottelat 2007a,b, Özuluğ and Freyhof 2007), based on a larger set of characters, discriminated 16 species of shemayas. They consider the following nominal taxa as valid in the Black Sea basin (clockwise from the Danube): *A. danubicus* Antipa, 1909 (anadromous), *A. mento* (Heckel, 1836) (landlocked), *A. sarmaticus* Freyhof & Kottelat, 2007b (anadromous and potamadromous), *A. mentoides* Kessler, 1859 (resident), *A. leobergi* Freyhof & Kottelat, 2007b (originally anadromous, also landlocked populations in dammed rivers), *A. derjugini* Berg, 1923 (resident), *A. istanbulensis* Battalgil, 1941 (resident), *A. schischkovi* (Drensky, 1943) (anadromous), and *A. mandrensis* (Drensky, 1943) (probably, landlocked). Shemayas distributed outside of the Black Sea basin include *A. volviticus* Freyhof & Kottelat, 2007a and *A. vistonicus* Freyhof & Kottelat, 2007a, both from lakes in eastern Greece, Aegean Sea basin (landlocked); two species from lakes in the Sea of Marmara basin in Turkey, *A. carinatus* Battalgil, 1941 (landlocked) and *A. nicaeensis* Battalgil, 1941 (landlocked, possibly extinct); two species in the Aegean Sea basin in western Turkey, *A. attalus* Özuluğ & Freyhof, 2007 (resident) and *A. battalgilae* Özuluğ & Freyhof, 2007 (resident), and *A. chalcoides* from the Caspian Sea basin (anadromous).

However, this taxonomic scheme still does not answer all remaining questions for the group. For example, it is not clear which species occurs between the ranges of *A. derjugini* and *A. istanbulensis* (e.g., in Sakarya, Kızılırmak, and Yesilirmak rivers). It is still to be clarified if there is (was) a single species in the Çoruh as supposed by Berg (1923) or there occurred two species or morphs, resident and anadromous as found by Deryugin (1899). Furthermore, the identification of shemaya in coastal lakes and limans in Bulgaria and Romania, other than Lake Madra, remains unknown.

Current knowledge of the morphology of the Black Sea shemayas is inadequate as the keys offered to distinguish them (Kottelat and Freyhof 2007) are often not helpful since most character ranges overlap. This may be the reason why a taxonomic compromise was offered (Parin et al. 2014) to consider only two shemaya species as valid, the Caspian *A. chalcoides* and the Black Sea *A. mento*.

As to the Danube drainage, as indicated above, Freyhof and Kottelat (2007b), based on literature and their own data, assumed an occurrence at least in former times, of two sympatric anadromous shemayas, one lake-dwelling species landlocked in a number of subalpine lakes, and one probable resident river species. The latter was only known from the Kolpa [Kupa] River and only three specimens were available for examination for Freyhof and Kottelat (2007b: 223). These authors found the Kolpa shemaya morphologically similar to *A. sarmaticus*, but differing in the number of gill rakers (27 vs. 28−34 in 28 individuals from the South Bug and the lower Danube). A morphological analysis of additional material from the Kolpa is important in resolving the taxonomy of the Danubian shemayas. Herein, we examine morphological variation of shemayas and provide a critical review of their historical distributions in the Danube.

## Material and methods

The triangular-shaped symphisis of the lower jaws is referred as the chin. The ventral keel is defined as the distance between the base of the anus and the level of the posterior ends of the pelvic-fin bases. The dorsal-fin insertion is the posterior-most point where the last dorsal-fin ray connects with the body. All measurements were made point-to-point with a dial caliper and recorded to the nearest of 0.1 mm. Methods for counting rays, lateral-line scales and scales along the scaleless part of the ventral keel, and for most measurements follow Kottelat and Freyhof (2007). Additional measurements of the cranium, jaws, and operculum as defined in Tables 1 and 3 were made point to point from the anteriormost extremity to the posteriormost extremity (lengths), from the uppermost extremity to the lowermost extremity (depths), and between the lateralmost extremities (widths). Length of the cranial roof was measured from the anterior margin of the supraethmoid to the base of the supraoccipital crest. Vertebral counts are given according to Naseka (1996). Standard length was measured from the tip of the upper jaw to the posterior margin of the hypurals. Head length was measured from the anteriormost extremity of the head (either tip of the upper jaw or the projected symphysis of the lower jaw) to the posterior opercular margin that includes the skin fold. In total, 49 morphometric indices were used for descriptions and statistical analyses as in Tables 1 and 3. These also included the length of the scaleless portion of the ventral keel relative to the keel length (Bogutskaya et al. 2010, fig. 1) and length of a gill raker at the junction of the lower and the upper limbs of the first gill arch (the 6−8th, usually the 7th, gill raker) relative to the opposite outer gill filament (Kottelat and Freyhof 2007, fig. 42). The last two rays in the dorsal and anal fins based on a single pterygiophore were counted as 1½ rays. Numbers of vertebrae and fin rays were counted from radiographs. In total, 16 meristic characters were examined (nine as given in Table 2 and numbers of branched dorsal-fin rays, branched pelvic-fin rays, intermediate vertebrae, total lateral scales, total lateral-line scales, scales above the lateral line, and scales below the lateral line). All characters were obtained from specimens of both sexes and combined in analyses and tables.

Abbreviations: SL, standard length; HL, lateral head length; HDBI, Croatian Biological Research Society; MNCN, Museo Nacional de Ciencias Naturales (Madrid, Spain); NMW, Naturhistorisches Museum Wien (Vienna, Austria); vs., versus; ZM NASU, Zoological Museum of National Academy of Sciences of Ukraine.

Cluster Analysis (CA), Multidimensional Scaling (MDS), Principal Component Analysis (PCA), Discriminant Function Analysis (DFA), and a Kruskal-Wallis test which is helpful for comparison of three and more groups with a presumably non-parametric distribution of variables, were performed using STATISTICA 6.0 and PRIMER v6.1.9 to identify the most important characters that contribute to the differentiation of samples and visualise the degree of morphological separation among the new species, *A. mento*, *A. sarmaticus*, and *A. leobergi*.

## Results

### Alburnus sava sp. n.

Taxon classificationAnimaliaCypriniformesCyprinidae

http://zoobank.org/AEE0CFF9-6F12-4DA6-BCF6-A4B6C61B3961

Figs 1 , 2 , 3a

#### Holotype.

MNCN 291345, 173.6 mm SL, female, Kolpa River at Griblje (45.58°N 15.30°E), Slovenia, 3 Oct 2013, coll. B. Levai.

#### Paratypes.

MNCN 291346-53, 8, 105−151.5 mm SL, same data as holotype; HDBI 255, 218 mm SL, Kupa [Kolpa] River at Ozalj (45.62°N 15.47°E), Sept 2011, Croatia, coll. D. Jelić; HDBI 1224, 3, 62.9−79.8 mm SL, same data as HDBI 255.

#### Diagnosis.

*Alburnus sava* sp. n. is distinguished from all other species of *Alburnus* in the Danube drainage by having 23−27, usually 24−26, gill rakers; the ventral keel usually completely scaled (scaleless maximum 15% of the keel length); 15−16 (mode = 15) branched pectoral-fin rays; the length of gill raker 65−70% of the length of the opposite outer gill filament; and a relatively long lower jaw (37−40% HL, 112−130% interorbital width).

#### Description.

**Figure 1.**
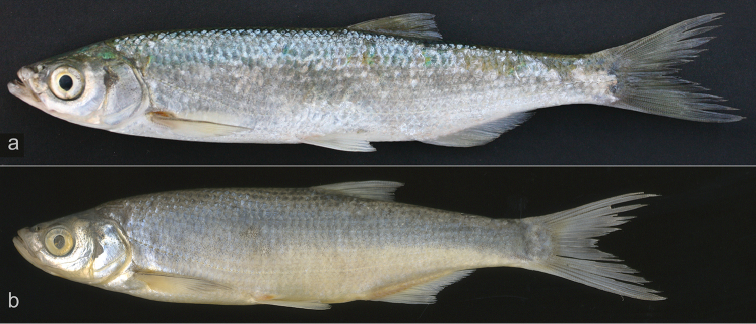
*Alburnus sava* sp. n., **a** MNCN 291345, holotype, 173.6 mm SL, before preservation **b** MNCN 291345, paratype, 151.5 mm SL, formaldehyde-preserved specimen.

The general appearance of *Alburnus sava* sp. n. can be seen in Figure 1. Relative measurements are provided in Table 1. Variation in ten (of the 16) examined meristic characters is provided in Table 2. The largest specimen, a spent female, is 218 mm SL. As the examined samples are rather small in number of specimens and contain individuals with a wide range in SL, Table 1 also presents the range and mean for the holotype and size groups separately. Body depth at the dorsal-fin origin in the 218 mm-long specimen represented 27% SL and considerably exceeded the range in body depth of smaller specimens (20−23% SL). The same is found for the depth of the caudal peduncle, 10% SL (60% length of caudal peduncle or 1.7 times in its length) vs. 9% (46−54% length of caudal peduncle or 1.9−2.2 times in its length), respectively. However, head length and eye diameter are clearly negatively allometric. The head length mean in the small-size group (63−80 mm SL) is 25.8% SL vs. 22.7% SL in specimens 174 and 218 mm SL; in smaller specimens the head length considerably exceeds the body depth while in the largest specimen it is much smaller than the latter. The eye diameter mean is 7.5% SL (29% HL) in the small-sized group (63−80 mm) vs. 5.1% SL (22.5% HL) in 218 mm long specimen.

**Table 1.** Morphometric data of *Alburnus sava* sp. n. Most influential characters (as discussed in text) given in bold.

	MNCN 291345, holotype	MNCN 291345, holotype and paratypes, n=9				HDBI 255	HDBI 1224, small-sized, n=3				All speciemens of *A. sava* sp. n., n=13			
		min	max	mean	sd		min	max	mean	sd	min	max	mean	sd
SL, mm	173.6	105	173.6	131.3		218	62.9	79.8	70.9		62.9	218		
**Body depth at dorsal-fin origin (% SL)**	21.3	19.9	23.3	21.4	1.1	26.6	20.7	21.5	21.2	0.4	19.9	26.6	21.8	1.7
Depth of caudal peduncle (% SL)	9.0	9.0	9.2	9.1	0.1	9.8	8.9	9.1	9.0	0.1	8.9	9.8	9.1	0.2
**Depth of caudal peduncle (% length of caudal peduncle)**	50.9	45.9	53.1	50.8	2.5	59.9	48.1	54.0	51.1	3.0	45.9	59.9	51.6	3.4
Body width at dorsal-fin origin (% SL)	9.8	9.7	11.6	10.5	0.7	11.6	7.6	8.6	8.2	0.5	7.6	11.6	10.1	1.3
Caudal peduncle width (% SL)	5.1	4.8	5.6	5.2	0.3	3.5	2.6	3.1	2.8	0.3	2.6	5.6	4.5	1.1
**Predorsal length (% SL)**	56.4	54.1	57.4	56.0	1.0	58.3	56.6	57.9	57.1	0.7	54.1	58.3	56.5	1.1
**Postdorsal length (% SL)**	36.5	34.3	38.4	35.6	1.2	35.3	34.2	35.1	34.5	0.6	34.2	38.4	35.3	1.1
Prepelvic length (% SL)	48.2	46.3	49.3	48.5	1.0	47.8	49.4	50.8	49.9	0.8	46.3	50.8	48.8	1.1
Preanal length (% SL)	71.3	67.5	71.3	69.5	1.1	69.6	68.5	71.1	69.6	1.3	67.5	71.3	69.5	1.1
**Pectoral – pelvic-fin origin length (% SL)**	25.8	23.1	25.8	24.4	0.9	27.7	24.5	27.1	25.4	1.5	23.1	27.7	24.9	1.3
Pelvic – anal-fin origin length (% SL)	21.7	20.5	23.9	21.7	1.0	24.1	19.2	21.1	20.1	0.9	19.2	24.1	21.5	1.4
**Caudal peduncle length (% SL)**	17.7	16.9	19.5	18.0	0.8	16.4	16.7	18.6	17.7	1.0	16.4	19.5	17.8	0.9
Dorsal-fin base length (% SL)	10.8	9.0	11.2	10.2	0.7	11.4	9.5	10.8	10.1	0.6	9.0	11.4	10.3	0.7
Dorsal-fin depth (% SL)	15.6	15.6	17.7	17.0	0.6	18.2	17.7	18.4	18.0	0.4	15.6	18.4	17.3	0.7
Anal-fin base length (% SL)	16.4	16.4	18.1	17.0	0.5	17.7	16.1	17.8	17.0	0.8	16.1	18.1	17.1	0.6
Anal-fin depth (% SL)	12.3	9.8	12.4	11.7	0.8	13.6	13.5	14.1	13.9	0.3	9.8	14.1	12.3	1.2
Pectoral-fin length (% SL)	17.7	17.7	21.5	20.1	1.2	20.6	20.7	21.1	21.0	0.2	17.7	21.5	20.3	1.1
Pelvic-fin length (% SL)	14.4	14.4	16.0	15.3	0.5	15.2	14.9	15.6	15.3	0.4	14.4	16.0	15.3	0.5
Head length (% SL)	22.7	22.7	24.8	23.8	0.7	22.7	25.4	26.4	25.8	0.5	22.7	26.4	24.2	1.2
**Head length (% body depth)**	106.7	99.0	122.5	111.2	7.4	85.3	117.9	124.3	122.1	3.6	85.3	124.3	111.7	11.1
Head depth at nape (% SL)	15.4	15.3	16.3	15.7	0.4	16.5	14.8	16.7	15.5	1.0	14.8	16.7	15.7	0.6
Head depth at nape (% HL)	67.8	61.7	69.4	66.1	2.7	72.7	56.5	65.6	59.9	5.0	56.5	72.7	65.1	4.6
Head depth through eye (% HL)	47.2	45.0	52.6	48.8	2.3	54.4	41.8	48.6	45.4	3.4	41.8	54.4	48.4	3.3
Maximum head width (% SL)	10.7	10.7	11.9	11.3	0.3	11.6	11.0	11.4	11.2	0.2	10.7	11.9	11.3	0.3
Maximum head width (% HL)	47.2	45.7	51.5	47.4	1.9	51.2	42.3	44.7	43.2	1.3	42.3	51.5	46.7	2.8
Snout length (% SL)	6.7	6.6	7.5	6.9	0.3	6.1	7.4	7.9	7.7	0.3	6.1	7.9	7.0	0.5
Snout length (% HL)	29.7	27.4	30.4	28.9	0.9	26.9	28.9	30.7	29.9	0.9	26.9	30.7	29.0	1.1
Eye horizontal diameter (% SL)	5.7	5.7	6.7	6.3	0.4	5.1	7.2	7.7	7.5	0.3	5.1	7.7	6.5	0.7
**Eye horizontal diameter (% HL)**	25.3	24.1	28.0	26.4	1.3	22.5	27.2	30.3	29.0	1.6	22.5	30.3	26.7	2.1
**Eye horizontal diameter (% interorbital width)**	78.7	78.7	90.6	83.7	4.0	66.6	87.6	94.1	91.0	3.2	66.6	94.1	84.1	7.1
Postorbital distance (% HL)	50.6	46.9	51.3	49.2	1.3	52.2	44.7	46.4	45.7	0.9	44.7	52.2	48.6	2.2
Interorbital width (% SL)	7.3	7.3	7.8	7.5	0.2	7.6	8.2	8.3	8.2	0.1	7.3	8.3	7.7	0.4
Interorbital width (% HL)	32.1	30.0	32.7	31.5	0.9	33.7	31.1	32.4	31.9	0.7	30.0	33.7	31.8	1.0
Length of upper jaw (% HL)	28.0	27.8	30.6	29.1	1.0	29.1	28.2	29.9	29.0	0.9	27.8	30.6	29.1	0.9
Length of upper jaw (% SL)	6.4	6.4	7.3	6.9	0.3	6.6	7.4	7.6	7.5	0.1	6.4	7.6	7.0	0.4
Length of lower jaw (% SL)	8.9	8.9	9.6	9.2	0.3	8.5	9.9	10.2	10.0	0.2	8.5	10.2	9.3	0.5
Length of lower jaw (% HL)	39.1	37.2	39.8	38.6	1.0	37.6	37.6	40.3	38.9	1.4	37.2	40.3	38.6	1.1
**Length of lower jaw (% interorbital width)**	121.8	116.6	129.6	122.7	4.4	111.6	119.3	125.3	121.9	3.1	111.6	129.6	121.6	4.9
**Length of lower jaw (% depth of operculum)**	98.2	96.7	107.6	100.9	3.9	90.7	102.3	109.6	107.1	4.2	90.7	109.6	101.5	5.6
Length of lower jaw (% cranium roof length)	63.8	59.7	67.1	62.5	2.8	62.4	62.7	64.0	63.2	0.7	59.7	67.1	62.8	2.2
**Length of lower jaw (% cranium width between margins of pterotics)**	99.9	94.4	104.6	99.1	3.2	85.8	100.0	100.5	100.2	0.3	85.8	104.6	98.4	4.6
Cranium roof length (% SL)	13.9	13.6	15.9	14.7	0.9	13.7	15.5	16.3	15.9	0.4	13.6	16.3	14.9	1.0
**Cranium width between margins of pterotics (% cranium roof length)**	63.9	59.5	68.6	63.2	3.0	72.7	62.7	63.7	63.1	0.5	59.5	72.7	63.9	3.6
**Cranium width between margins of sphenotics (% cranium roof length)**	55.2	49.0	57.3	53.8	2.4	65.6	56.9	60.0	58.9	1.8	49.0	65.6	55.9	4.2
Cranium width between margins of lateral ethmoids (% cranium roof length)	19.7	19.7	23.4	21.8	1.4	23.4	16.6	18.0	17.3	0.7	16.6	23.4	20.9	2.4
**Cranium width between margins of lateral ethmoids (% cranium width between margins of pterotics)**	30.9	30.9	38.4	35.0	1.9	32.1	26.6	28.6	27.5	1.0	26.6	38.4	32.7	3.6
Depth of operculum (% HL)	39.9	36.3	39.9	38.3	1.4	41.5	35.3	36.8	36.3	0.9	35.3	41.5	38.1	1.8
***Ratios***														
Interorbital width/eye horizontal diameter	1.3	1.1	1.3	1.2	0.1	1.5	1.1	1.1	1.1	0.0	1.1	1.5	1.2	0.1
Snout length/eye horizontal diameter	1.2	1.0	1.2	1.1	0.1	1.2	1.0	1.1	1.0	0.1	1.0	1.2	1.1	0.1
Head depth at nape/eye horizontal diameter	2.7	2.3	2.7	2.5	0.1	3.3	1.9	2.2	2.1	0.1	1.9	3.2	2.5	0.3
Head length/caudal peduncle depth	2.5	2.5	2.8	2.6	0.1	2.3	2.8	2.9	2.9	0.1	2.3	2.9	2.6	0.2
Length of caudal peduncle/caudal peduncle depth	2.0	1.9	2.2	2.0	0.1	1.7	1.9	2.1	2.0	0.1	1.7	2.2	1.9	0.1
Length of lower jaw/caudal peduncle depth	1.0	1.0	1.1	1.0	0.0	0.9	1.1	1.1	1.1	0.0	0.9	1.1	1.0	0.1
Pectoral fin length/pectoral – pelvic-fin origin distance	0.7	0.7	0.9	0.8	0.1	0.7	0.8	0.9	0.8	0.1	0.7	0.9	0.8	0.1
Predorsal length/head length	2.5	2.3	2.5	2.4	0.1	2.6	2.1	2.3	2.2	0.1	2.1	2.6	2.3	0.1

The mouth is upturned and the mouth cleft is straight. The tip of the mouth is about at a level with the upper margin of the pupil. The lower jaw is long, its length 112−130% interorbital width. The chin is variably developed (Fig. 1). The holotype has a well developed chin while the chin of the paratype is smoothed and slightly projected.

The ventral keel between the pectoral-fin bases and the anus is well pronounced but not sharp and usually completely covered by scales (in 8 specimens, including the holotype) or scaleless (exposed) for 1−2 scales only (Table 2), reaching up to 15% of the keel.

**Figure 2.**
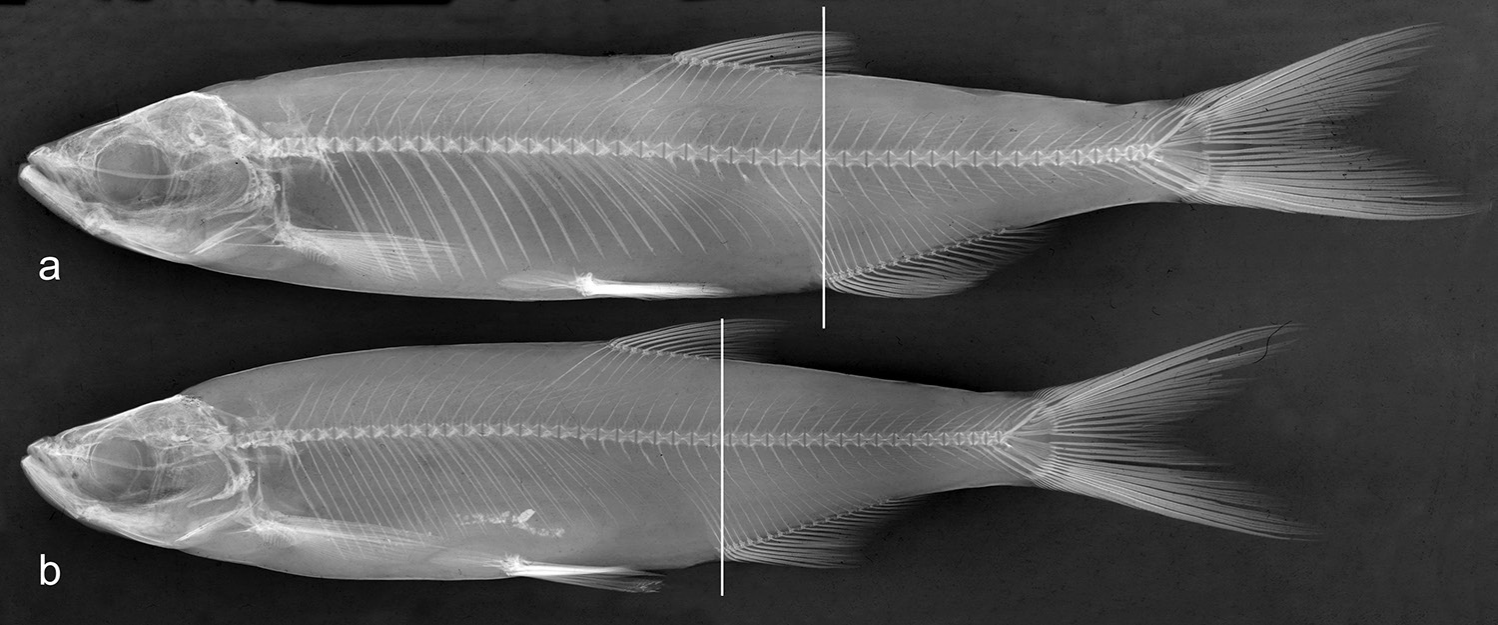
Radiograph of *Alburnus sava* sp. n., same specimens as in Fig. 1; vertical lines showing origin of anal fin located on (**b**) or slightly behind (**a**) vertical of dorsal-fin insertion.

Dorsal fin with 3 unbranched and 8½ branched rays. Anal fin with 3 unbranched and 15½ or 16½ branched rays (Table 2). Origin of anal fin located on (in three specimens) or slightly behind the vertical of the dorsal-fin insertion (Fig. 2).

**Figure 3.**
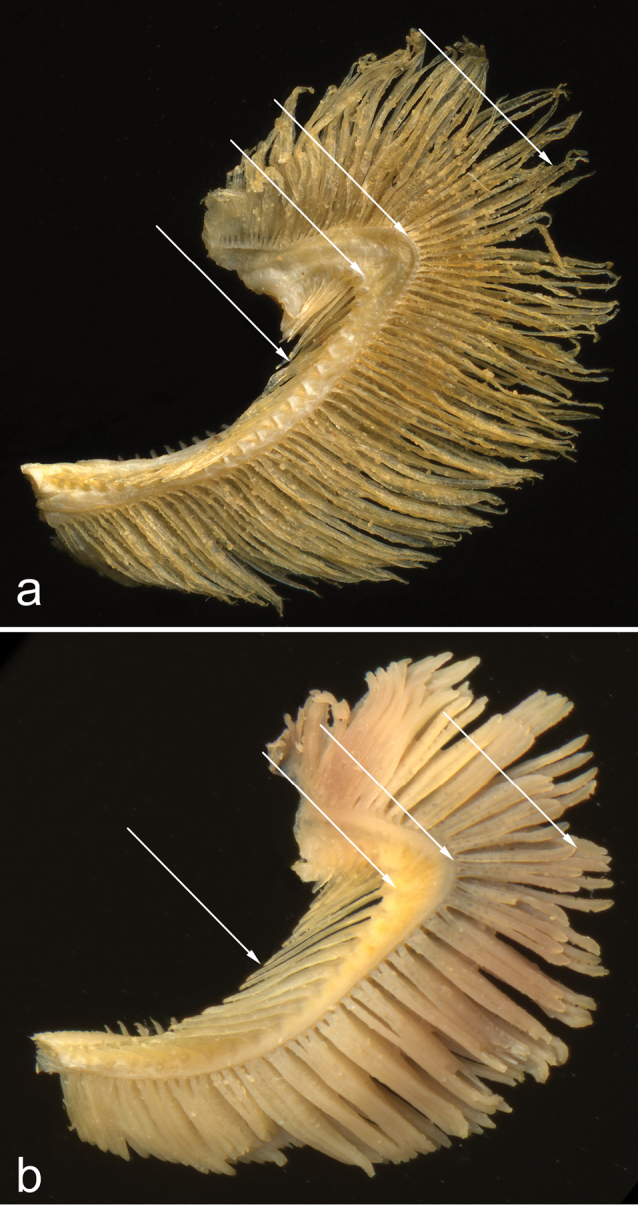
First gill arch in **a** *Alburnus sava* sp. n., HDBI 255, 218 mm SL (length of gill raker is 70% of opposite gill filament length) and **b** *A. mento*, NMW 79592, 184 mm SL (length of gill raker is 120% of opposite gill filament length).

Number of gill rakers 23−27, mode 24−26 (Table 2). In two specimens examined (80 and 218 mm SL) length of gill raker 65% and 70% (Fig. 3a) the length of the opposite gill filament in the outer row. Pharyngeal teeth 2.5-5.2 (n =2 paratypes).

Total lateral-line scales number (61)63−64, mode 63; lateral-line scales to the posterior margin of hypurals 57−62, mode 60. Total vertebrae 44−45, 23−24 abdominal and 20−21 caudal (Table 2; Fig. 2).

No nuptial tubercles in the examined material. Four dissected individuals were females. Overall colouration is silvery with no orange or red pigment at fin bases and no faint dark midlateral stripe in both freshly caught and preserved specimens.

**Table 2a.** Meristic data for *Alburnus sava* sp. n. and three species used for comparisons. Counts in holotype marked with *.

	Branched anal-fin rays						Branched pectoral-fin rays				
	13½	14½	15½	16½	17½	Mean (+½)	15	16	17	18	Mean
*A. sava* sp. n., n=13			4*	9		**15.7**	8*	5			**15.4**
*A. sarmaticus*, South Bug, n=5		1	3		1	**15.2**	2	3			**15.6**
*A. sarmaticus*, Danube, n=15		1	9	5		**15.3**	5	7	3		**15.9**
*A. leobergi*, n=6			2	3	1	**15.8**	1	5			**15.8**
*A. mento*, n=62 for anal-fin rays, n=50 for pectoral-fin rays	2	16	25	14	5	**15.1**	1	11	26	12	**17.0**

**Table 2b.** 

	**Predorsal abdominal vertebrae**					**Abdominal vertebrae**				**Caudal vertebrae**				**Total vertebrae**				
	**15**	**16**	**17**	**18**	**mean**	**23**	**24**	**25**	**mean**	**20**	**21**	**22**	**mean**	**43**	**44**	**45**	**46**	**mean**
*A. sava* sp. n., n=13		7*	6		**16.5**	8	5*		**23.4**	1*	12		**20.9**		9*	4		**44.3**
*A. sarmaticus*, South Bug, n=5		5			**16.0**	4	1		**23.2**	1	3	1	**21.0**	1	2	2		**44.2**
*A. sarmaticus*, Danube, n=15	1	9	5		**16.3**	7	7	1	**23.6**	5	8	2	**20.8**		10	4	1	**44.4**
*A. leobergi*, n=6		5		1	**16.3**	5	1		**23.2**		3	3	**21.5**		2	4		**44.7**
*A. mento*, n=60	2	35	22	1	**16.4**	30	27	3	**23.6**	7	31	22	**21.3**	1	20	29	10	**44.8**

**Table 2c.** 

	Lateral-line scales to posterior hypural margin														
	54	56	57	58	59	60	61	62	63	64	65	66	69	71	mean
*A. sava* sp. n., n=13			1	1	3*	5	1	1							**59.6**
*A. sarmaticus*, South Bug, n=5						2			1	2					**62.2**
*A. sarmaticus*, Danube, n=15		1	1	1	1	3	2	2	3		1				**60.7**
*A. leobergi*, n=6		1		1		2	1	1							**59.5**
*A. mento*, n=50	1		1	3	11	9	7	5	2	7		2	1	1	**61.1**

**Table 2d.** 

	Gill rakers																
	19	20	21	22	23	24	25	26	27	28	29	30	31	32	33	34	mean
*A. sava* sp. n., n=13					**1**	**4**	**3**	4*	**1**								**25.0**
*A. sarmaticus*, South Bug, n=5												**1**	**3**			**1**	**31.4**
*A. sarmaticus*, Danube, n=15										**1**	**3**	**3**	**3**	**4**	**1**		**30.6**
*A. leobergi*, n=6							**1**			**1**		**2**	**1**	**1**			**29.3**
*A. mento*, n=50	**1**	**1**	**1**	**8**	**11**	**15**	**10**	**2**	**1**								**23.6**

**Table 2e.** 

	Scales along scaleless portion of ventral keel														
	0	1	2	3	4	5	6	7	8	9	10	11	12	13	mean
*A. sava* sp. n., n=13	8*	**1**	**4**												**0.7**
*A. sarmaticus*, South Bug, n=5		**3**	**2**												**1.4**
*A. sarmaticus*, Danube, n=15		**1**	**5**	**5**	**2**	**2**									**2.9**
*A. leobergi*, n=6		**1**	**4**			**1**									**2.3**
*A. mento*, n=50				**1**	**5**	**7**	**6**	**9**	**11**	**3**	**3**	**2**	**2**	**1**	**7.2**

#### Distribution and habitat.

**Figure 4.**
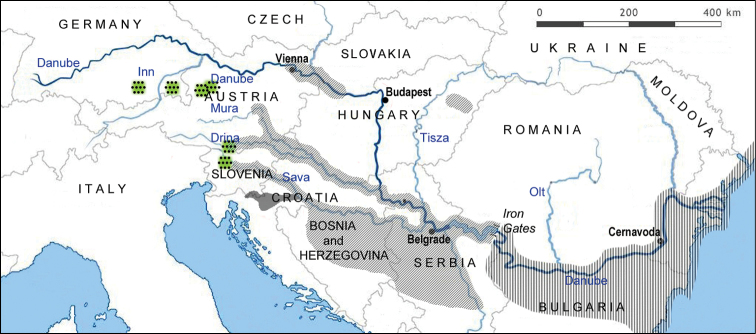
Map of distribution of shemayas in the Danube drainage: *A. mento*, *A. sava* sp. n., distribution of potamadromous shemaya (supposedly *A. sava* sp. n.), anadromous *A. sarmaticus* and/or *A. danubicus*.

The species is currently known from the Kolpa River drainage, a tributary of the Sava River in the upper Danube drainage, Black Sea basin (Fig. 4). *Alburnus sava* sp. n. is a potamadromous species, occurring in streams and rivers with moderate to rapid current and a gravel and cobble bottom; in spring, during the spawning season, the species migrates upstream to smaller tributaries to shallow riffles where they spawn.

#### Etymology.

The species name refers to the Sava River. A noun in apposition.

#### Vernacular name.

Local names are bucov or velika pliska in Croatian and Serbian, pegunica in Slovene.

### Comparisons

The new species belong to the *Alburnus mento* group of shemayas (former genus *Chalcalburnus*), which includes *A. mento*, *A. sarmaticus*, and *A. leobergi*, as defined by Freyhof and Kottelat (2007b) to have 57−62 lateral-line scales to the posterior margin of hypurals and 15−16½ branched anal-fin rays. The maximum size of *Alburnus sava* sp. n. in examined materials is 218 mm SL (adult female), which is about the body size of mature adults of migratory shemayas in the Black Sea basin, including the Danube − ca. 200−300 mm (vs. 100−130 mm in resident small-sized *A. derjugini* and *A. mentoides*) (Drensky 1943, Berg 1949, Tscherbukha 1965, Movchan and Smirnov 1983).

*Alburnus sava* sp. n. does not demonstrate a distinct difference in most morphometric characters from all or either of the other Danubian species and *A. leobergi* (Tables 1, 3; discussed below regarding statistical analysis). The same is true for numbers of vertebrae (total and in vertebral regions), branched anal-fin rays, and scales in the lateral row, total lateral line and the lateral line to the posterior margin of hypurals) (Table 2).

**Figure 5.**
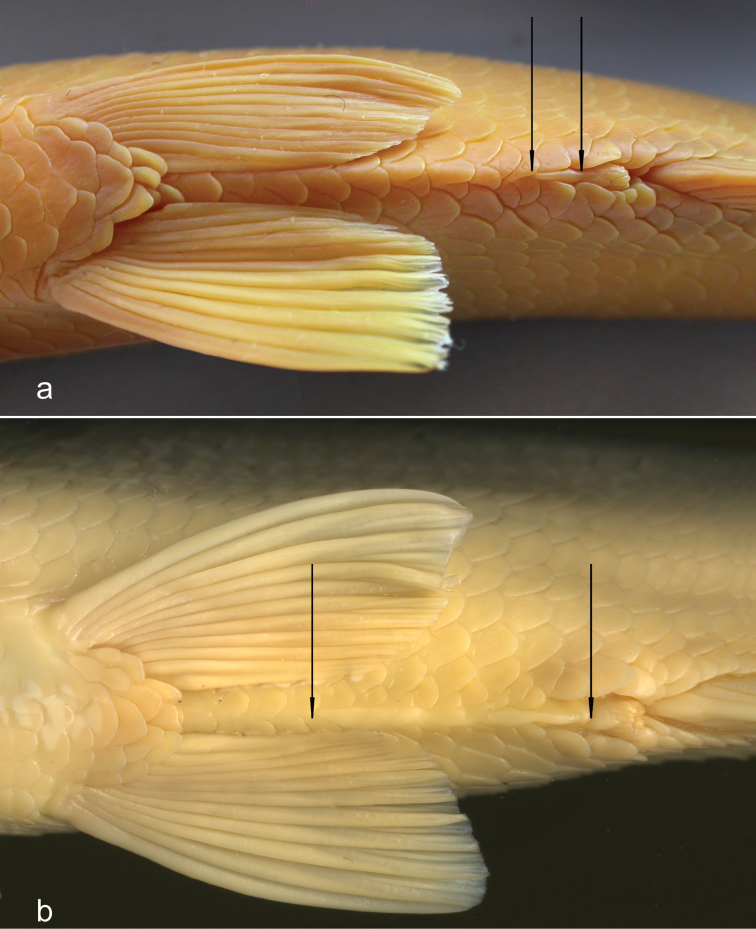
Ventral keel. Arrows showing anterior margin of anus base and beginning of scaleless portion of keel in **a** *Alburnus sarmaticus*, ZM NASU 4145, South Bug, 184.2 mm SL, 2 scales along scaleless portion of ventral keel; and **b** *A. mento*, NMW 79592, Mondsee, 135.1 mm SL, 11 scales along scaleless portion of ventral keel.

However, *Alburnus sava* sp. n. can be clearly distinguished from *A. sarmaticus* (Table 2) using two meristic characters. First, the number of gill rakers is 23−27 in *A. sava* sp. n. vs. 28−34 in *A. sarmaticus*; this same range for *A. sarmaticus* was found by Freyhof and Kottelat (2007b: table 3). A wider range in number of gill rakers, 26−33 (mean = 30), was reported by Tscherbukha (1965) for specimens from the South Bug. Second, the ventral keel of *A. sava* sp. n. is usually completely covered by scales, while in *A. sarmaticus* the typical state is a scaleless keel along about 10−15% its length (i.e., 1−3 scales exposed (Fig. 5a) found in 16 of 20 specimens). Among specimens of *A. sarmaticus* examined, neither a completely scaled keel nor a keel exposed along 6 scales (4−6 indicated by Freyhof and Kottelat (2007b) was found.

**Table 3.** Morphometric data for *Alburnus sarmaticus*, *A. mento*, and *A. leobergi*. Most influential characters (as discussed in text) given in bold.

	*Alburnus sarmaticus*, South Bug; n=5				*Alburnus sarmaticus*, Lower Danube and delta; n=15				*Alburnus mento*, Bavaria, Traunsee, Mondsee; n=10				*Alburnus leobergi*, NMW Yasenskiy Bay, Sea of Azov, n=6			
	min	max	mean	sd	min	max	mean	sd	min	max	mean	sd	min	max	mean	sd
SL, mm	170.7	204.5	184.5		157.5	209	183.2		134.3	204.5	147.9		154.8	193.1	176.6	
**Body depth at dorsal-fin origin (% SL)**	25.4	27.2	26.0	0.8	23.9	27.3	25.8	1.0	19.8	24.0	22.0	1.6	22.2	26.2	24.9	1.5
Depth of caudal peduncle (% SL)	9.2	10.3	9.5	0.5	8.8	10.5	9.6	0.4	7.9	9.7	9.0	0.6	9.5	10.1	9.7	0.2
**Depth of caudal peduncle (% length of caudal peduncle)**	48.3	61.0	53.8	6.0	49.3	64.9	58.4	4.8	41.5	59.2	51.0	5.9	57.4	75.8	64.1	7.9
Body width at dorsal-fin origin (% SL)	11.4	14.7	12.6	1.2	10.1	13.8	11.5	1.2	8.7	13.2	11.4	1.8	11.6	12.6	12.1	0.3
Caudal peduncle width (% SL)	3.2	4.9	3.8	0.7	3.4	4.7	4.1	0.4	3.4	8.7	6.1	2.4	3.8	4.7	4.2	0.3
**Predorsal length (% SL)**	53.5	55.8	54.3	1.0	52.7	57.3	55.8	1.1	52.8	57.2	55.5	1.5	55.6	59.7	57.0	1.5
**Postdorsal length (% SL)**	35.6	39.4	36.6	1.6	34.7	38.9	36.1	1.0	35.1	37.9	36.3	1.0	30.3	36.4	34.5	2.5
Prepelvic length (% SL)	44.5	49.4	47.8	2.0	46.4	49.0	47.8	0.7	46.1	50.6	48.2	1.6	46.3	50.4	47.5	1.5
Preanal length (% SL)	65.4	69.8	68.0	1.6	66.9	72.8	70.1	1.7	66.4	70.4	68.3	1.4	68.4	73.3	70.1	1.7
**Pectoral – pelvic-fin origin length (% SL)**	22.6	27.4	25.4	2.0	25.0	27.9	26.3	0.8	24.8	27.5	26.4	1.0	24.2	26.1	25.0	0.7
Pelvic – anal-fin origin length (% SL)	21.6	22.9	22.5	0.5	21.5	27.2	24.3	1.7	19.3	23.4	21.5	1.2	22.6	26.1	24.1	1.3
**Caudal peduncle length (% SL)**	16.5	19.0	17.9	1.1	14.6	18.9	16.6	1.3	16.2	19.8	17.7	1.2	13.3	16.9	15.3	1.6
Dorsal-fin base length (% SL)	11.7	12.1	11.9	0.2	9.6	12.4	11.1	0.7	9.0	11.6	10.6	0.7	10.4	11.7	11.0	0.5
Dorsal-fin depth (% SL)	17.8	21.6	19.1	1.5	16.5	20.5	18.5	1.4	14.8	17.4	16.1	0.8	17.9	21.4	19.0	1.3
Anal-fin base length (% SL)	18.4	19.4	18.7	0.4	16.3	18.7	17.6	0.8	14.2	19.6	17.1	1.6	17.2	19.1	17.8	0.7
Anal-fin depth (% SL)	11.3	14.0	12.8	1.0	10.3	15.0	13.1	1.3	10.2	12.5	11.2	0.7	12.3	14.4	13.3	0.7
Pectoral-fin length (% SL)	18.9	21.3	20.2	1.0	19.6	22.3	20.4	0.7	17.1	19.8	18.7	0.8	19.8	21.4	20.4	0.6
Pelvic-fin length (% SL)	14.6	16.6	15.4	0.8	14.3	17.3	15.4	0.7	14.0	15.5	14.6	0.5	15.0	16.2	15.6	0.5
Head length (% SL)	22.3	24.4	23.5	0.9	21.9	24.0	23.0	0.5	20.6	24.1	22.7	1.1	22.3	25.3	23.4	1.0
**Head length (% body depth)**	84.8	96.0	90.4	4.4	83.8	95.9	89.4	4.1	97.6	110.5	103.4	5.0	86.7	106.2	94.5	7.2
Head depth at nape (% SL)	13.9	15.8	15.0	0.7	14.3	16.4	15.4	0.5	14.0	16.4	15.1	0.9	15.3	16.5	15.9	0.5
Head depth at nape (% HL)	62.7	65.7	63.9	1.3	62.7	71.2	67.2	2.1	63.7	71.3	66.6	2.4	65.4	69.6	67.8	1.9
Head depth through eye (% HL)	45.8	51.8	47.8	2.3	46.6	51.2	49.2	1.4	45.8	52.9	48.4	2.2	47.3	51.5	49.9	1.5
Maximum head width (% SL)	10.6	11.7	11.4	0.5	10.5	12.1	11.2	0.5	10.1	12.4	11.2	0.9	11.1	11.9	11.4	0.3
Maximum head width (% HL)	47.5	50.8	48.4	1.4	44.2	52.8	48.8	2.5	46.2	52.6	49.4	2.1	45.4	51.4	48.9	2.2
Snout length (% SL)	6.6	7.2	6.9	0.2	6.0	6.8	6.4	0.2	5.6	7.6	6.5	0.7	6.0	6.9	6.6	0.3
Snout length (% HL)	28.1	30.7	29.4	1.3	26.9	29.2	27.6	0.8	26.2	32.1	28.3	1.9	25.6	29.6	28.0	1.5
Eye horizontal diameter (% SL)	5.3	6.0	5.6	0.3	5.0	6.7	5.7	0.5	4.9	6.0	5.6	0.4	4.6	5.5	5.0	0.3
**Eye horizontal diameter (% HL)**	23.1	24.5	23.8	0.6	22.1	29.0	24.7	2.2	23.0	27.0	24.8	1.2	20.7	22.0	21.4	0.6
**Eye horizontal diameter (% interorbital width)**	57.6	76.5	66.2	7.1	64.4	86.1	71.6	6.7	70.9	87.5	77.5	6.2	58.3	66.6	63.5	3.6
Postorbital distance (% HL)	49.2	51.4	50.2	0.9	47.5	53.8	50.8	1.5	46.2	52.0	48.7	1.7	49.4	52.8	51.6	1.2
Interorbital width (% SL)	7.8	9.3	8.5	0.6	7.2	8.5	7.9	0.4	6.7	7.9	7.3	0.4	7.5	8.4	7.9	0.4
Interorbital width (% HL)	32.0	40.2	36.2	2.9	32.1	37.2	34.5	1.5	29.5	33.6	32.0	1.2	32.7	35.5	33.7	1.2
Length of upper jaw (% HL)	23.8	27.1	25.2	1.5	24.4	28.7	26.9	1.1	24.6	29.8	27.4	1.6	23.9	27.2	26.3	1.2
Length of upper jaw (% SL)	5.5	6.6	5.9	0.4	5.6	6.6	6.2	0.3	5.3	6.9	6.2	0.6	5.4	6.8	6.2	0.4
Length of lower jaw (% SL)	7.7	9.1	8.4	0.5	8.1	8.9	8.4	0.3	7.7	9.2	8.5	0.6	7.8	9.0	8.4	0.4
Length of lower jaw (% HL)	33.8	37.6	35.6	1.5	34.6	38.2	36.6	1.0	36.3	38.8	37.4	0.9	34.3	37.3	35.8	1.3
**Length of lower jaw (% interorbital width)**	89.2	106.5	98.8	7.4	96.6	113.4	106.5	5.7	111.8	124.0	116.9	3.6	103.9	112.8	106.4	3.4
**Length of lower jaw (% depth of operculum)**	89.0	102.1	97.3	5.6	89.0	104.7	97.2	4.5	93.2	114.4	100.6	7.1	87.1	93.9	91.4	2.8
Length of lower jaw (%cranium roof length)	55.3	67.8	61.1	4.8	59.7	73.5	64.9	3.5	63.2	73.8	68.0	3.9	60.1	68.3	63.9	2.7
**Length of lower jaw (% cranium width between margins of pterotics)**	79.3	90.4	85.7	4.5	83.2	93.3	88.8	3.1	86.8	96.5	90.9	3.6	85.1	91.0	88.5	2.6
Cranium roof length (% SL)	12.6	14.9	13.7	1.0	12.1	13.8	13.0	0.5	11.7	13.8	13.0	4.4	12.5	14.1	13.1	0.7
**Cranium width between margins of pterotics (% cranium roof length)**	69.6	76.2	71.3	2.8	69.3	84.2	73.1	3.6	66.1	77.6	72.0	0.7	69.4	75.4	72.3	2.7
**Cranium width between margins of sphenotics (% cranium roof length)**	60.5	70.4	63.8	3.9	62.4	78.2	67.3	3.9	53.6	65.1	59.0	4.0	64.5	68.7	66.7	1.6
Cranium width between margins of lateral ethmoids (% cranium roof length)	20.5	23.3	22.6	1.2	21.2	27.0	23.9	1.7	17.7	23.0	20.4	1.9	21.8	26.5	24.0	1.6
**Cranium width between margins of lateral ethmoids (% cranium width between margins of pterotics)**	29.4	33.4	31.7	1.8	29.7	39.0	32.8	2.3	23.7	31.0	27.4	3.2	29.2	36.0	33.2	2.8
Depth of operculum (% HL)	33.6	40.8	36.7	2.6	34.4	40.2	37.7	1.4	34.0	39.7	37.3	1.9	36.9	40.7	39.2	1.4
***Ratios***																
Interorbital width/eye horizontal diameter	1.3	1.7	1.5	0.2	1.2	1.6	1.4	0.1	1.1	1.4	1.3	0.1	1.5	1.7	1.6	0.1
Snout length/eye horizontal diameter	1.1	1.3	1.2	0.1	0.9	1.3	1.1	0.1	1.0	1.3	1.1	0.1	1.2	1.4	1.3	0.1
Head depth at nape/eye horizontal diameter	2.6	2.8	2.7	0.1	2.2	3.0	2.7	0.2	2.4	2.8	2.7	0.1	3.0	3.3	3.2	0.1
Head length/caudal peduncle depth	2.2	2.6	2.5	0.1	2.2	2.6	2.4	0.1	2.4	2.8	2.5	0.1	2.3	2.5	2.4	0.1
Length of caudal peduncle/caudal peduncle depth	1.6	2.1	1.9	0.2	1.5	2.0	1.7	0.1	1.7	2.4	2.0	0.2	1.3	1.7	1.6	0.2
Length of lower jaw/caudal peduncle depth	0.8	0.9	0.9	0.1	0.8	0.9	0.9	0.0	0.9	1.1	1.0	0.0	0.8	0.9	0.9	0.0
Pectoral fin length/pectoral – pelvic-fin origin distance	0.7	0.9	0.8	0.1	0.7	0.8	0.8	0.0	0.7	0.8	0.7	0.0	0.8	0.9	0.8	0.0
Predorsal length/head length	2.2	2.4	2.3	0.1	2.3	2.5	2.4	0.1	2.3	2.6	2.4	0.1	2.3	2.6	2.4	0.1

Though measurements based on the limited material examined cannot be used for taxonomic purposes with regard to being variable depending on season, sex, size and other factors, some relative measurements may have some taxonomic value as reflecting basic morphological differences between these species. *Alburnus sava* sp. n. differs from *A. sarmaticus* by a longer upper jaw, 28−31% HL (vs. 24−29% HL); a longer lower jaw, 37−40% HL or 112−130% interorbital width (vs. 34−38% HL or 89−113% interorbital width); and a longer cranial roof, 14−16% SL (vs. 12−15% SL) (Tables 1, 3).

Freyhof and Kottelat (2007b) described *A. sarmaticus* as having 17½ or fewer branched anal-fin rays as an anadromous shemaya in the Danube; they described *A. danubicus*, a purportedly extinct species from the Danube, as having 17−20½ branched anal-fin rays; this was based on their opinion of data of Antipa (1909) and Drensky (1943) for an anadromous shemaya from the lower Danube. However, no specimens have been reported since then with 18½ or more branched anal-fin rays and some authors (Halasi-Kovacs and Harka 2012, Stefanov and Trichkova 2015) continue to use *danubicus* for the Danubian shemaya as the only anadromous species in the Danube. Materials collected by Shishkov [=Chichkoff] and Drensky, still deposited in the National Museum of Natural History in Sofia (Bulgaria), should be examined to clarify the problem.

When compared to *A. mento*, *A. sava* sp. n. with 15½ or 16½ branched anal-fin rays do not differ from the former species possessing 13−18½ branched anal-fin rays (Table 2, Fig. 7; Freyhof and Kottelat 2007b). Three more counts are overlapping and only statistically (see below) different in *Alburnus sava* sp. n. and *A. mento* . First, the new species has 23−27, usually 24−26, gill rakers (vs. 19−27, usually 22−25 in *A. mento*, Table 2). Second, 8½ branched dorsal-fin rays (vs. 7−8½, 7½ found in 14 from 62 examined specimens; *A. mento* is the only Danubian species with often 7½ branched dorsal-fin rays). Third, 15−16, with a mode of 15, branched pectoral-fin rays that is the lowest count among the examined samples (Table 2) (vs. the highest count, (15)16−18, mode = 17, found in *A. mento*).

**Figure 6.**
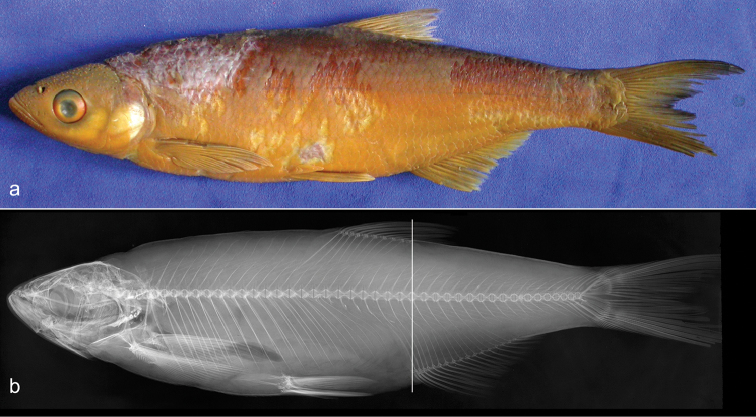
*Alburnus sarmaticus* ZM NASU 4145, South Bug, 184.2 mm SL; general appearance (**a**) and radiograph (**b**). Vertical line showing origin of anal fin located on vertical of dorsal-fin insertion.

**Figure 7.**
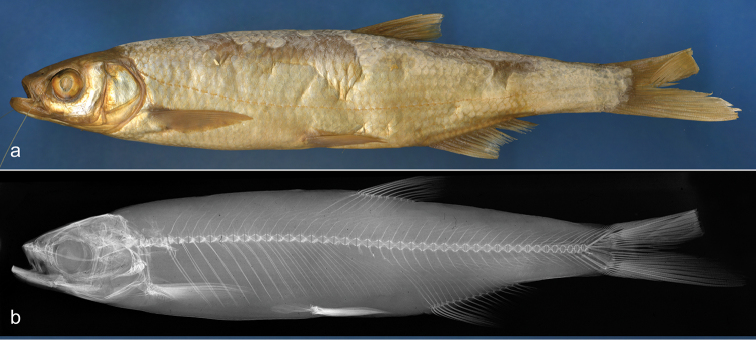
*Alburnus mento* NMW 55629, general appearance and radiograph, Kremsmünster, 144 mm SL.

*Alburnus sava* sp. n. and *A. mento* are distinguished in a much clearer way by the length of the scaleless portion of the ventral keel and the relative length of a gill raker. As shown by Freyhof and Kottelat (2007a, b), these characters have a considerable taxonomic value in the genus *Alburnus* . In *A. sava*, the ventral keel is completely scaled or there is a very short scaleless portion of the keel which is only 1 or 2 scales long, maximum 15% of the keel length (the shortest keel among the examined species, Table 2). In contrast, *A. mento* has a very long scaleless portion of the keel which is 3 to 13, usually 4−12, scales long (Fig. 5b) that is 25−85% of the keel length (up to 90% according to Freyhof and Kottelat (2007b).

In *A. sava* sp. n. (Fig. 3a), the length of the gill raker is 65−70% of the opposite outer gill filament. In *A. sarmaticus*, it is similar, 50−93%, usually 60−75%, averaging 68% (examined in 20 specimens). In contrast, in *A. mento* (Fig. 3b), the length of the gill raker is 75−126%, usually 90−120%, averaging 103%, of the length of the opposite gill filament (examined in 20 specimens).

*Alburnus sava* sp. n. further differs from *A. mento* by the shape of the symphysial part of the jaws. Neither a pronounced chin nor a knob formed by the symphysis of the lower jaws were found in the new species. In contrast, *Alburnus mento* of a different size have a very prominent chin and a strong knob on the lower jaw entering a corresponding notch formed at the symphysis of the upper jaws, a feature also typical of the asp *Leuciscus aspius*.

*Alburnus sava* sp. n. differs from geographically close *A. mandrensis*, *A. schischkovi*, and *A. istanbulensis* (data from Freyhof and Kottelat (2007a) and Özuluğ and Freyhof (2007) by having the ventral keel completely scaled or exposed for 1 or 2 scales (vs. 6−12) and 15−16½ branched anal-fin rays (vs. 13−15½). The new species further differs from *A. mandrensis* by the length of the gill raker 65−70% of the opposite outer gill filament (vs. 45−55%) and 23−27, usually 24−26, gill rakers (vs. 25−35) and from *A. schischkovi* by having 57−62 lateral-line scales to posterior margin of hypurals (vs. 63−67). *Alburnus sava* sp. n. is also distinguished from *A. istanbulensis* by having the length of the gill raker 65−70% of the opposite outer gill filament (vs. 30−55%) and 23−27, usually 24−26, gill rakers (vs. 24−35, usually 26−32).

**Note on syntypes of *A. mento*.** A comment should be given clarifying the status of the specimens labelled as syntypes of *A. mento* in NMW. The original description (Heckel 1836: 225−226) clearly indicates three samples of specimens it was based on. All three samples are still present among specimens labeled as syntypes in NMW. The set of Acquisition Sheets (bound in a number of books at present) is the most reliable source of primary original information which accompanied the samples at the moment of their accession in the collection.

1. Specimens collected by Heckel in September 1824 in Lake Traun («... at Gmunden, .. especially abundant under the Traun bridge»). Heckel’s specimens collected in September 1824 are registered under the acquisition number 1824.II.10: «Traun, … Heckels Reise durch Oberösterreich… Nr. 80». Two specimens are still in NMW (16261 and 16441), and one was sent to the Paris Museum. The two NMW specimens have a standard length (139.8 mm and 134 mm, respectively) which corresponds to a total length equaling “Spanne” [Handspanne] (the distance between the end of the little finger and the end of the thumb that is about 18−22 cm), mentioned in the original description.

2. Specimens later [than 1824] received from Agassiz. These specimens are most probably those registered under the acquisition number 1830.II.3. The acquisition 1830.II contains 7 entries in total (e.g., 1830.II.1 is for *Gobio uranoscopus*) and reads «Bavaria. November 1829. Von Herrn Leopold Fitzinger durch Kauf». This acquisition is made by Jos. Natterer and 1830.II.3 refers to *Leuciscus macroramphus* Agassiz with the name *Aspius Heckelii* Fitz. handwritten later by Heckel. “4-6” Individual: (?) 4 were sent somewhere on exchange [in Tauch]. The labels for NMW 50440, 55650, and 55652 (with the acquisition number 1830.II.3) reading «Durch Agassiz aus München» appeared later, at Steindachner’s time, and are most probably based on information from the Heckel’s description of *Aspius mento* Agassiz as a synonym of *Aspius heckelii* Fitzinger (Heckel 1836: 225): «Später erhielt das hiesige Museum durch die Güte des Herrn Professor Agassiz sehr schöne Exemplare seines *Aspius Mento* aus München; ich habe nun diese Exemplare auf das sorgfältigste mit jenen aus der Traun verglichen, …» [«Later, the local museum received by the generosity of Professor Agassiz very beautiful specimens of his *Aspius Mento* from Munich; I have now most carefully compared these specimens with those from the Traun...»]. So, the exact locality is not given but Bavaria in a general way, and this does not exclude Bavarian lakes, e.g., Starnberger See, closest to Munich in the south-wets still inhabited by *A. mento*.

3. One specimen (9 Viennese inches long) from the Danube near Vienna. This specimen was registered under the acquisition number 1836.I.19: «Danube at Vienna. November 1835».

The accession information for one more sample labeled as syntypes, NMW 55629, does not match the original description. It contains four specimens registered under the acquisition numbers 1835.VI.1 (2 specimens) and 1835.VI.1a (2 specimens) which say that the specimens were received by exchange from [Dr] Schreibers in 1832 and 1834, respectively, from near Stift Kremsmünster (Kremsmünster Abbey; in the town of Kremsmünster in the district of Kirchdorf an der Krems, Upper Austria, 29 km from Gmunden on Traunsee). However, the source of the acquisition may mean any water body in the surroundings of Kremsmünster where an astronomical and geophysical observatory (“Mathematical tower”), the first in the world, was located which included a natural history museum with botanical and zoological collections (still existing as a weather station). However, the label (from Steindachner’s time) says «Traunsee» (probably just based on the original description). As Kremsmünster was not mentioned in the original description by Heckel (1836), even if the specimens are from Lake Traun, they do not belong to the Traunsee sample collected by Heckel in 1824. So, the NMW 55629 specimens do not belong to the type series and the designation of the 150 mm long (SL) specimen from this sample as lectotype by Freyhof and Kottelat (2007b: 217) is invalid.

### Statistical analysis

**Figure 8.**
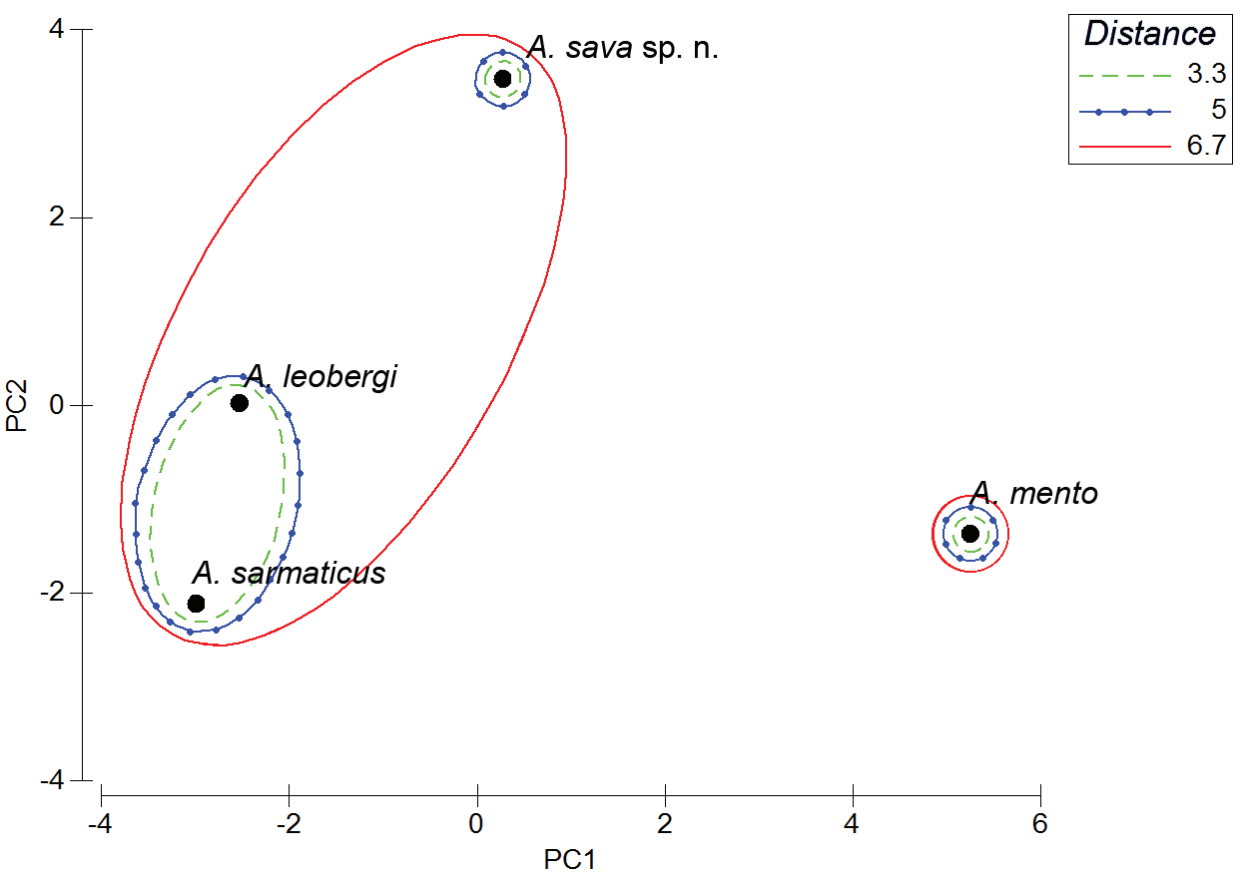
Results of PCA and MDS (Complete Linkage, Euclidean distances) realised in Primer v6 on meristic characters of *Alburnus sava* sp. n., *A. sarmaticus*, *A. mento*, and *A. leobergi*.

Results of PCA and MDS analyses applied to 16 meristic variables outlined three clusters (Fig. 8). *A. sava* sp. n. is most distant from *A. mento. Alburnus sarmaticus* and *A. leobergi* are comparatively close; specimens of these two species cannot be distinguished based on their meristic characters.

Based on the PCA, the most influential variables are the number of gill rakers and the number of scales along the scaleless portion of the ventral keel−these variables’ contributions based on covariances are over 0.24 (Factor 1: 0.668885, Factor 2: 0.316944 and Factor 1: 0.241753, Factor 2: 0.434431, respectively) vs. less than 0.1025 for all other variables. A Kruskal-Wallis test revealed four (including the two mentioned above) characters different on a statistically significant (0.01%) level: the number of scales above the lateral line (H (3, 48)=18.0313487 p=0.0004); the number of branched pectoral-fin rays (H (3, 48)=19.2995932 p=0.0002); the number of gill rakers (H (3, 48)=34.8078209 p=0.0000001), and scales along the scaleless portion of the ventral keel (H (3, 48)=29.1477545 p=0.00002).

**Figure 9.**
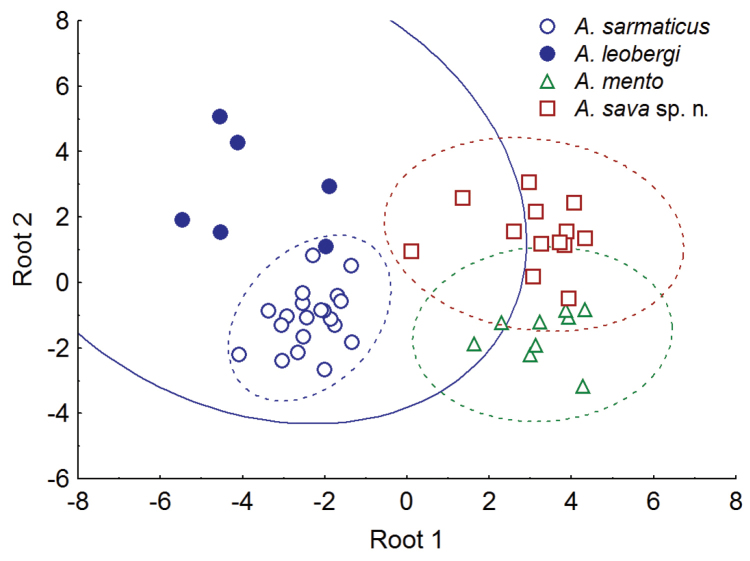
Result of DFA carried out on morphometric characters to discriminate *Alburnus sava* sp. n., *A. sarmaticus*, *A. mento*, and *A. leobergi*.

To reduce the number of morphometric indices (47 in total, as in Tables 1 and 3) to be used in a DFA, a Kruskal-Wallis test was applied and 10 characters were excluded as not significantly different (p>0.01%) between the samples. Among other 37 characters, a PCA revealed 15 variables whose contribution to either of Factors 1 and 2 or both was the highest (over an arbitrary fixed threshold of 0.01 based on the number of the used variables and their variance); these characters are given in bold in Tables 1 and 3. These 15 indices were used for a DFA (Fig. 9) that revealed three groups corresponding to *A. sava* sp. n., *A. mento*, and *A. sarmaticus* + *A. leobergi*.

**Figure 10.**
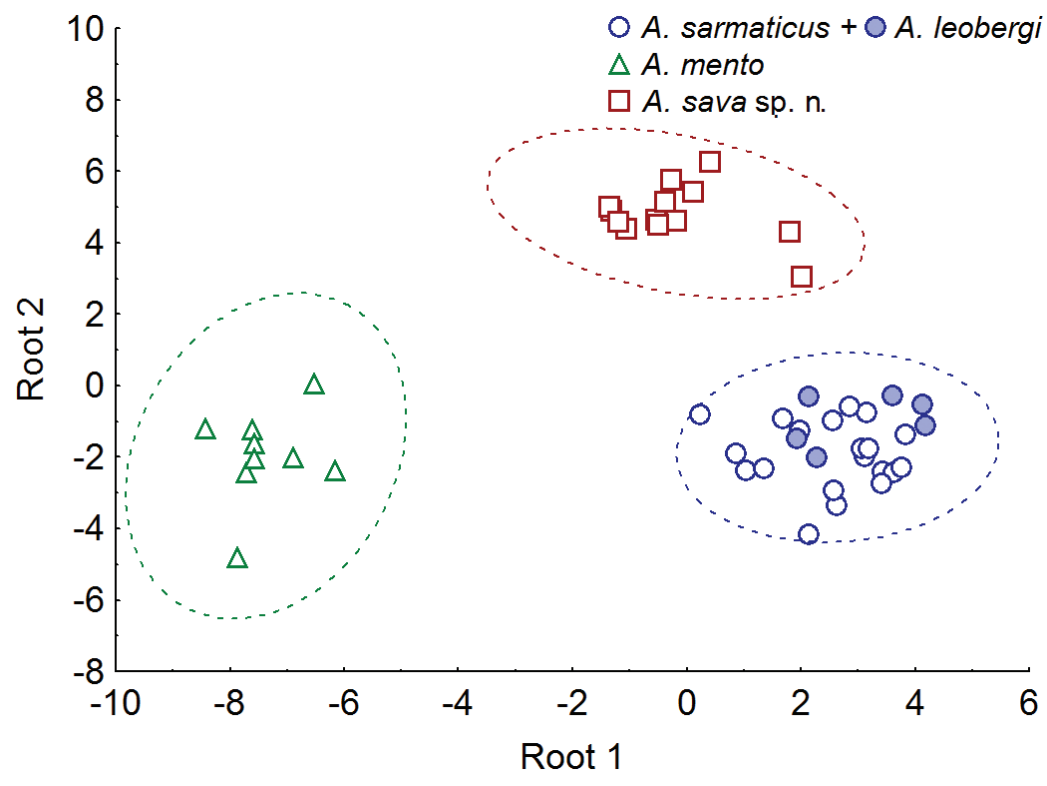
Result of DFA performed on 19 (4 meristic and 15 morphometric) most contributing characters to discriminate *A. sava* sp. n., *A. mento*, and *A. sarmaticus* + *A. leobergi*.

As a further step, the samples of *A. sarmaticus* and *A. leobergi* were combined as being indistinguishable by the studied morphometric and meristic characters (Figs 8, 9) and a DFA was performed based on 19 most contributing characters (4 meristic and 15 morphometric ones as discussed above) (Fig. 10); predicted classifications for *A. sava* sp. n., *A. mento*, and *A. sarmaticus* + *A. leobergi* were 100%-correct. DFA statistics values are as follows: Wilks’ Lambda 0.00618, approx. F (38, 54) =16.661, p<0.0000, which indicate almost perfect discrimination. *Alburnus sava* sp. n. is the most distant from *A. mento* (Squared Mahalanobis Distance equals 103.99); *A. sarmaticus+A. leobergi* is much closer (54.11) but still significantly distant. The most removed, by the characters considered, are *A. mento* and *A. sarmaticus+A. leobergi* (107.02). Partial Lambdas demonstrate the unique contribution of the respective variable to the discriminatory power of the whole model, and the most significant (Partial Lambda <0.9) for discrimination of the samples under consideration were number of scales along scaleless portion of ventral keel (0.481705), cranium width between margins of sphenotics (% cranium roof length) (0.545483), cranium width between margins of pterotics (% cranium roof length) (0.803940), number of gill rakers (0.810206), number of branched pectoral-fin rays (0.830673), number of scales above lateral line (0.845142), length of lower jaw (% interorbital width) (0.878840), and body depth at dorsal-fin origin (% SL) (0.886565).

### Discussion on distribution of shemayas in the Danube and adjacent areas

Shemayas are reported in the Danube from the upper reaches down to the delta (Fig. 4).

In the upper Danube, a shemaya, commonly identified as *A. mento*, was known to occur (or still occurs) in lakes in Germany in systems of the Isar (Starnberger See [Würmsee]) and the Inn (Chiemsee, Simsee, and Waginger See), in Austria in the Traun River system (Traunsee, Attersee, Mondsee, Hallstättersee, Grundsee, Wolfgangsee, probably Fuschlsee and Irrsee) and the Drava [Drau] system (Wörthersee [Vrba]), and in Slovenia in the upper Sava (Bled [Veldeser See, Blejsko Jezero]) (Freyhof and Brunken 2004, Freyhof and Kottelat 2007b, Glowacki 1896, Heckel 1836, Honsig-Erlenburg and Petutschnig 2002, Patzner et al. 1993). Most of these lakes belong to Danubian tributaries on the northern slope of the Alps. Lake Wörthersee is located much further south, on the northern slope of the Karawankas mountain ridge (one of the south-eastern ridges of the Alps) and belongs to the Gurk [Krka] River in the upper Drava system. *Alburnus mento* was found in the lake, in the inflowing Reifnitzbach and the outflowing Glanfurt River (Honsig-Erlenburg and Petutschnig 2002). Freyhof and Kottelat (2007b) found no morphological difference between *A. mento* inhabiting Lake Wörthersee and *A. mento* in the lakes of the Isar and Inn river systems. This means that *A. mento* has a rather geographically fragmented range and it cannot be excluded that a shemaya which was historically present in Lake Bled (Glowacki 1896, Munda 1926) in the very upper reach of the Sava River belonged to *A. mento*. No museum samples of the Bled shemaya are known to exist to check this hypothesis. The lake morph (lake phenotype) from different lakes likely have a polyphyletic origin as found for lake *Coregonus* (e.g., Kottelat 1997, Douglas et al. 1999, Østbye et al. 2006). However, a problem of distinguishing between single (homology) and multiple (homoplasy of ecomorphological traits) origins of the distinct lake phenotype (= *A. mento*) and its taxonomic implication is beyond the scope of this paper.

In the Danube in Germany, Austria, Hungary, and Croatia downstream to the confluence with the Drava [Drau], one collection specimen (now lost) was known from near Vienna (NMW 55630, a syntype); this specimen may represent the anadromous or potamadromous Danubian species rather than the landlocked *A. mento* (Berg 1932, Freyhof and Kottelat 2007b). We examined the other NMW specimen labeled as *A. mento* from the Danube at Lobau near Vienna (NMW 54831) but it is a *Leuciscus aspius*. Shemayas were also known to be found occasionally in the Hungarian section of the Danube downstream of the Austrian section (Hankó 1931) but are extirpated (Halasi-Kovacs and Harka 2012). It was also mentioned as a fish occurring in a lake in the Maros [Mureș] river system (a tributary of the Tisza) (Herman 1887).

Based on records of Glowacki (1896) and Munda (1926), most subsequent authors gave the upper Sava and the upper Drava as areas of historical distribution of shemaya (e.g., Taler 1953, Sabioncello 1967, Povž 2012, Povž et al. 2015). Povž and Sket (1990) supposed its occurrence in the Mura [Mur] (a tributary of Drava), the entire Drava, and the lower course of the Sava. Povž et al. (1998) presented a first reliable record (with voucher specimens and a photo) of a shemaya from the Sava system. Several specimens were collected in the Kolpa [Kupa] River, a right tributary of the Sava, between Dol and Sodevci on August 11, 1996, between Dečina and Radenci on September 10, 1996, and up and below the Radenci dam on September 11, 1996.

In Croatia, a shemaya is reported from the Drava, Sava, Kupa [Kolpa] and their tributaries (Bogut et al. 2006, Mrakovčić et al. 2006, Jelić et al. 2016) but no voucher specimens are known except those from the Kolpa. However, it cannot be excluded that the authors based their statements on reliable data from local fishery sources. Further south and eastwards, in Bosnia and Herzegovina, a shemaya is found in the lower Sava and its tributaries Una, Sana, Vrbas, Ukrina, Tinja, Bosna (upper section with tributaries and from Žepče to the confluence with the Sava), and Drina (at Ljubovija and from Loznica to the confluence with the Sava) (Vuković and Ivanović 1971, Vuković 1977, 1982, Kosorić et al. 1980, Hađiselimović and Hamzić 1999, Mikavica and Savić 1999, Drešković et al. 2011, Simonović et al. 2015, Jelić et al. 2016). In Serbia, it is reported from the Danube down to the Iron Gates [Đerdap] at the border with Romania (Janković 1996, Simonović 2001) and from the Zapadna Morava River between the Ovčar Banja and Međuvršje reservoirs (Veljović et al. 1985) and in its tributary Ibar (Šorić 2009).

Formally, the name *A. sava* sp. n. is applied herein only for the shemaya found in the Kolpa but we suppose that this potamadromous fish inhabits (or inhabited in the past) the entire Sava River system and, probably, the Danube upstream from the Iron Gates being geographically separated from the anadromous shemaya of the lower Danube (*A. danubicus* and *A. sarmaticus*).

Right below the Iron Gates (a 134-m-long gorge on the main stream of the Danube dammed in 1964 and 1977), shemaya were known in Romania (Bănărescu 1964) and the entire Bulgarian stretch of the Danube with tributaries (e.g., Iskyr, Vit, Osym, Yantra) where it still occurs though rare (numerous publications reviewed by Stefanov and Trichkova (2015). Records of shemaya in the lower section of the Danube (downstream from Cernavodă) in Romania and Ukraine were also numerous (e.g., Drensky 1943, Bănărescu 1961, 1964, Movchan and Smirnov 1983).

So, historically, shemaya in the Danube were distributed in almost the entire drainage. The length of the Danube and its major tributaries makes it reasonable to suppose that the drainage was historically populated by an anadromous shemaya (entering the river for spawning from the sea) and a resident potamadromous shemaya. At present, it is difficult to assume how far the anadromous form(s) or species used to migrate upstream in the Danube. All anadromous shemayas in the Black Sea and the Sea of Azov basins spend most of the year foraging in coastal sea areas, estuaries, and limans with salinity up to 10−12‰ and wintering in deep places of lower reaches of rivers. Before damming of rivers, they migrated upstream to reach spawning grounds at a distance from tens to hundreds of kilometers depending on the size of the river and availability of grounds suitable for spawning (riffles with gravel bottom and rapid flow). Shemaya were known to migrate up to Pervomaysk in the South Bug, the Dnieper River Rapids (upstream of the town of Zaporizhia) in the Dnieper, and the Oskol River in the Siversky Donets system of the Don River (Movchan and Smirnov 1983). It looks quite likely that the anadromous shemaya in the main Danube migrated upstream to at least the Iron Gates gorges. Our data support a taxonomic conclusion of previous authors (Bănărescu 1961, 1964, Tscherbukha 1965, Loshakov 1973, Freyhof and Kottelat 2007b) that the South Bug and Dnieper and the Danube are populated by one and the same species.

No materials were available for us to clarify a border between the ranges of *A. danubicus*, *A. sarmaticus*, and *A. sava* sp. n. This question can only be solved when specimens from the mainstream Sava and its tributaries in Croatia, Bosnia and Herzegovina, and Serbia are studied as well as specimens from the Danube below its confluence with the Sava. This task cannot be easily implemented because of the rarity of the fish; though, in some tributaries of the Sava in Bosnia and Herzegovina it is still often recorded (Jelić et al. 2016).

### Comparative material

*Alburnus sarmaticus* ZM NASU 4145, 5, 170.7−204.5 mm SL, Ukraine, South Bug River, 2 km downstream of Yuzhno-Ukrainsk, 7−11.05.1989; ZM NASU 2529, 6, 157.5−209 mm SL, Ukraine, Danube, 1922; NMW55507, 3, 178−208.8 mm SL, Lower Danube, Galatz, 1910; NMW55509, 5, 169.4−202.8 mm SL, Lower Danube, Cernavodă, 1910; NMW 55654,1, 175.3 mm SL, Delta of Danube, Vilkovo, 08.1924.

*Alburnus leobergi* NMW 6, 154.8−193.1 mm SL, Yasenskiy Bay, Sea of Azov.

*Alburnus mento* NMW 16261, 1, syntype, 139.8 mm SL, Traunsee; NMW 16441, 1, syntype, 134 mm SL, Traunsee; 50440, 1, syntype, 220.4 mm SL, Bavaria; NMW 50441, 1, 89.2 mm SL, Wörther-See; NMW 55628, 7, 123.8−147.4 mm SL, Gilgen, Wolfgangsee; NMW 55629, 2, 129.2−144 mm SL, Kremsmünster; NMW 55634, 17, 103.3−156.4 mm SL, Traunsee; NMW 55636, 9, 97.1−122.7 mm SL, Attersee; NMW 55642, 4, 148−162.9 mm SL, Gilgen, Wolfgangsee; NMW 55646, 1, 104.4 mm SL, Wörther See; NMW 55648, 2, 112,2−112.9 mm SL, Attersee; NMW 56652, 1, syntype, 210.9 mm SL, Bavaria; NMW 79592, 5, 134.3−143.3 mm SL, Mondsee; NMW 79593, 1, 214.3 mm SL, Mondsee; NMW 80138, 1, 221.2 mm SL, Mondsee; NMW 80622, 1, 191.2 mm SL, Mondsee; NMW 80623, 7, 170.4-220.5 mm SL, Mondsee.

### Key to *Alburnus* species in the Danube drainage

**Table:** 

1	Ventral keel between pectoral bases and anus completely scaleless. Lateral-line scales to posterior margin of hypurals 42−50	***A. alburnus***
–	Ventral keel between pectoral bases and anus completely scaled or covered by scales at least at anterior part of keel. Lateral-line scales to posterior margin of hypurals 52−71	**2**
2	Branched anal-fin rays 17−20½	***A. danubicus***
–	Branched anal-fin rays 13−17½, usually 14−16½	**3**
3	Gill rakers 28−34	***A. sarmaticus***
–	Gill rakers 19−27	**4**
4	Branched pectoral-fin rays 15−16, usually 15. Ventral keel between pectoral bases and anus scaleless along 1−2 scales (up to 15% of keel length) or completely scaled	***A. sava***
–	Branched pectoral-fin rays (15)16−18, usually 17. Ventral keel between pectoral bases and anus scaleless along 3−13 scales (25−90% of keel length)	***A. mento***

## Supplementary Material

XML Treatment for Alburnus sava
